# Cross-disease genetic and epigenetic architecture of the *MOBP* locus shows convergence in ALS-PSP

**DOI:** 10.64898/2026.03.25.714147

**Published:** 2026-03-27

**Authors:** Katherine Fodder, Megha Murthy, Rohan de Silva, Towfique Raj, Kurt Farrell, Jack Humphrey, Conceição Bettencourt

**Affiliations:** 1. Department of Neurodegenerative Disease, UCL Queen Square Institute of Neurology, London, UK; 2. Department of Clinical and Movement Neurosciences, UCL Queen Square Institute of Neurology, London, UK; 3. Reta Lila Weston Institute, UCL Queen Square Institute of Neurology, London, UK; 4. Department of Genetics and Genomic Sciences, Icahn Genomics Institute, Estelle and Daniel Maggin Department of Neurology, Icahn School of Medicine at Mount Sinai, New York, NY, USA.; 5. Nash Family Department of Neuroscience, Icahn School of Medicine at Mount Sinai, New York, NY, USA; 6. Ronald M. Loeb Center for Alzheimer’s Disease, Icahn School of Medicine at Mount Sinai, New York, NY, USA; 7. Friedman Brain Institute, Icahn School of Medicine at Mount Sinai, New York, NY, USA; 8. Department of Pathology, Icahn School of Medicine at Mount Sinai, New York, NY, USA; 9. Department of Artificial Intelligence & Human Health, Icahn School of Medicine at Mount Sinai, New York, NY, USA; 10. Neuropathology Brain Bank & Research CoRE, Icahn School of Medicine at Mount Sinai, New York, NY, USA

## Abstract

Myelin oligodendrocyte basic protein (*MOBP*) is an abundant oligodendrocyte gene implicated in multiple neurodegenerative diseases. Genetic variation at the *MOBP* locus has been associated with risk for progressive supranuclear palsy (PSP), amyotrophic lateral sclerosis (ALS), frontotemporal lobar degeneration (FTD), corticobasal degeneration (CBD), Alzheimer’s disease (AD), Lewy body dementia (LBD), and Creutzfeldt–Jakob disease (CJD). Epigenetically, *MOBP* promoter hypermethylation and reduced expression have been reported in multiple system atrophy (MSA). Although *MOBP* is thought to play a role in oligodendrocyte morphology and myelin structure, how genetic and epigenetic variation at this locus influences gene regulation and contributes to disease risk remains poorly understood across neurodegenerative disorders.

Here, we investigated whether shared or disease-specific genetic mechanisms at *MOBP* converge on altered DNA methylation and expression across neurodegenerative disorders. We analysed *MOBP* variants using summary statistics from recent GWAS for ALS, PSP, FTD, LBD, PD, MSA, AD, and CJD. Colocalisation (COLOC and SuSiE-coloc) was used to test whether disease-associated variants overlapped between diseases, and with oligodendrocyte expression quantitative trait loci (eQTLs) and bulk brain methylation quantitative trait loci (mQTLs). To further investigate mQTL effects at this locus, rs1768208, a variant previously associated with PSP, was genotyped in an overlapping brain methylation cohort, allowing direct testing of genotype–methylation associations in frontal white matter tissue.

ALS and PSP GWAS demonstrated strong association at *MOBP*, with most strongly associated SNPs (e.g. rs631312, rs616147, rs1768208) shared between both disorders. Colocalisation analyses indicated high posterior probability that ALS and PSP share the same causal variant, with weaker overlap with FTD. mQTL colocalisation highlighted cg15069948, located near an exon junction within *MOBP*, as strongly colocalising with the ALS/PSP risk variants. In complementary tissue analyses, rs1768208-T carriers showed hypomethylation at cg15069948 in PSP brains. No genotype-methylation effects were detected in MSA or Parkinson’s disease.

Together with prior evidence of promoter hypermethylation and reduced expression in MSA, our findings identify cg15069948 as a regulatory methylation site linking ALS/PSP risk variants to altered *MOBP* methylation, and support *MOBP* dysregulation as a shared feature of neurodegeneration. However, the underlying mechanisms appear disease-specific, highlighting the complexity of involvement of this gene across neurodegenerative disorders.

## Introduction

Although neurodegenerative diseases are traditionally defined as distinct entities, considerable overlap in clinical features and neuropathology is commonly observed(1). Consistent with this, genetic, transcriptomic, and epigenetic studies increasingly implicate shared molecular pathways across subsets of these disorders. Genome-wide association studies (GWAS) have revealed both shared and disease-specific risk loci. Defining where their genetic architectures converge or diverge is crucial for uncovering common pathways of neurodegeneration and identifying therapeutic targets with potential cross-disease relevance.

Myelin-associated oligodendrocyte protein (MOBP), which is almost exclusively expressed in oligodendrocytes (2) is linked to multiple neurodegenerative diseases through distinct mechanisms. Although its exact function remains unclear, MOBP is the third most abundant CNS myelin protein and is thought to stabilise myelin and support oligodendrocyte morphological differentiation (3,4). Functional studies by Schäfer et al. demonstrated that *MOBP* knockdown reduces surface area of MBP-positive oligodendrocytes and impairs myelin-like membrane formation (5).

Genetic variation in *MOBP* is associated with the risk of multiple neurodegenerative diseases, underscoring its biological relevance. Risk-associated SNPs have been identified in progressive supranuclear palsy (PSP)(6–10), corticobasal degeneration (CBD) (10), frontotemporal dementia (FTD)(11), amyotrophic lateral sclerosis (ALS)(12), and in APOE-ε4–positive Alzheimer’s disease (13). One key variant, rs1768208-T, an intronic SNP in *MOBP*, is robustly associated with PSP and CBD risk and with white-matter degeneration and faster executive decline in bvFTD(14). Although its functional impact is not fully understood, the risk allele has been associated with increased *MOBP* expression in PSP brain tissue(15,16).

DNA methylation studies in MSA have identified *MOBP* as a key dysregulated locus. The *MOBP* promoter has been reported as the most differentially methylated region in MSA cerebellar white matter, with similar changes also observed in frontal and occipital white matter(17). The presence of these changes in mildly affected regions suggests they occur early in disease and may contribute to pathogenesis. *MOBP* expression was reduced in cerebellar white matter and in oligodendrocytes of MSA compared to controls (18). Follow-up analyses showed that *MOBP* promoter hypermethylation is inversely correlated with *MOBP* expression (2), consistent with DNA methylation driving *MOBP* downregulation. At the protein level, MOBP isoform abundance and ratios were unchanged in the white matter of MSA, whereas PSP showed increased expression of all three isoforms and a shift toward larger isoforms(2). MOBP shows disease-specific localisation in cerebellar white matter, restricted to myelin in controls but enriched in glial cytoplasmic inclusions in MSA, where it interacts with α-synuclein (2). Sequestration into Lewy bodies has also been reported in PD and LBD, although this remains controversial (19).

Given our previous work and the involvement of *MOBP* in common and rare neurodegenerative diseases, understanding shared mechanisms involving this gene in disease processes could give crucial insight into common pathways that lead to neurodegeneration and provide targets for therapeutic intervention in a broader range of diseases. Here, we investigate how this gene is dysregulated across several neurodegenerative diseases through investigations of genetic, epigenetic and transcriptomic data. We identify a strong signal of shared genetic association between PSP and ALS suggestive of same causal SNPs that is not observed in the other neurodegenerative diseases. Methylation analysis identified a CpG site (cg15069948) at the *MOBP* locus that colocalises with the PSP/ALS genetic signal, providing evidence for a shared regulatory mechanism linking genetic risk to epigenetic variation at this locus.

## Methods

### Datasets (GWAS, mQTL, and eQTL)

To investigate genetic architecture at the *MOBP* locus across neurodegenerative diseases, we performed colocalisation analyses using publicly available GWAS summary statistics. For each disease, we obtained full GWAS summary statistics and accompanying lists of genome-wide significant loci from the respective studies (Table 1). When required, missing fields were harmonised as follows: Standard errors were derived from reported effect sizes and P-values; minor allele frequencies were supplemented using European reference data from the 1000 Genomes Project; and SNP coordinates or RS IDs were matched using Ensembl GRCh37/hg19(20).

Summary statistics from methylation quantitative trait loci (mQTL) analysis were provided by the study authors (*N* = 534)(27). Oligodendrocyte specific expression quantitative trait loci (eQTL) summary statistics were made available by Bryois et al. (28) and downloaded from https://zenodo.org/records/7276971. For all the analyses, we used a region comprised between 1 Mb upstream and downstream of the coordinates *of MOBP* (GRCh37 Chromosome 3: 39,508,689–39,570,970) to investigate association between disease QTLs, mQTLs and eQTLs.

### Co-localisation Analysis

As an initial exploratory step, we assessed the similarity of association patterns across neurodegenerative diseases and molecular traits at the *MOBP* locus using correlation-based approaches. First, we calculated pairwise correlations between SNP-level GWAS *p*-values across diseases within the *MOBP* region (±1 Mb around the gene) to evaluate the extent of shared genetic signal.

Next, using bulk brain mQTL summary statistics, we extracted data for all CpG sites within ±1 Mb of *MOBP* and, for each CpG, computed SNP-wise correlations between *p-values* for mQTL disease-associated GWAS SNPs across the locus. This analysis was used to identify CpGs whose genetic regulation showed concordant patterns with disease association.

An analogous approach was applied to eQTL data, where SNP-wise correlations were calculated between disease GWAS *p*-values and oligodendrocyte-enriched eQTL *p*-values within the same genomic window, providing an initial indication of shared genetic architecture between disease risk and *MOBP* expression.

We next performed formal colocalisation analysis using the COLOC R package (version 3.2–1)(29). COLOC evaluates whether two association signals, such as disease GWAS signals and mQTL or eQTL effects, are consistent with a shared causal variant within a defined genomic region. For each disease GWAS dataset, we extracted all SNPs within the *MOBP* locus (±1 Mb around the gene). Prior probabilities in COLOC reflect assumptions about the likelihood that a SNP is associated with trait 1 (p1), trait 2 (p2), or both traits (p12). As only a small proportion of SNPs are expected to be causal, default priors are typically low (p1 = p2 = 1×10^−^; p12 = 1×10^−^ ). However, given prior evidence supporting association at the *MOBP* locus, we increased these values (p1 = p2 = 1×10^−3^; p12 = 1×10^−^ ) to reflect a higher prior probability of association and colocalisation in this targeted analysis. Approximate Bayes Factors (ABFs) were computed internally by COLOC to estimate posterior support for each hypothesis. To complement the COLOC ABF framework and to allow for multiple causal variants within a region, we applied the SuSiE (30) fine-mapping model to both the GWAS and QTL datasets. SuSiE identifies credible sets of putative causal SNPs by modelling associations as the sum of multiple independent effects, returning a posterior inclusion probability (PIP) for each SNP and one or more credible sets.

### Genotyping of *MOBP* rs1768208

Several SNPs within *MOBP* have been associated with neurodegenerative diseases, with rs1768208 repeatedly reported as a lead variant. We therefore selected rs1768208 (Chr3:39,508,968, GRCh37) for targeted genotyping in a subset of samples with available DNA methylation data.

A PCR 381 bp fragment encompassing the variant was amplified using flanking primers (forward: 5′-TCC TCT CAA GCC TCA AAC TCT C-3′; reverse: 5′-GGC AAC TCA GCC CAG AAA TTT G-3′). Each 15 μl PCR reaction contained 7.5 μl Promega GoTaq^®^ Green Master Mix, 2.25 μl of each primer (3 μM), 2 μl genomic DNA (~100 ng), and 2 μl dH O, and was amplified using a standard touchdown protocol. PCR products were verified by agarose gel electrophoresis and purified with an ExoSAP mix (exonuclease I and FastAP).

Sanger sequencing was performed by Source Bioscience using the forward primer, and chromatograms were visualised in Benchling (https://benchling.com/) to determine rs1768208 genotypes.

**Fig. 1 |:**
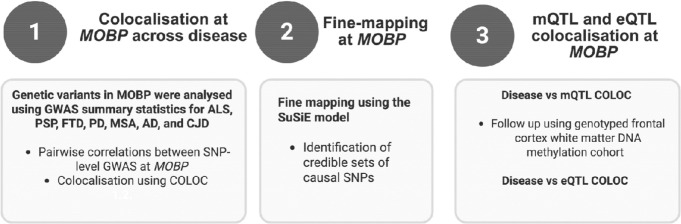
Schematic overview of the multi-step analytical strategy used to characterise genetic and regulatory mechanisms at the *MOBP* locus. 1) Cross-disease colocalisation: GWAS summary statistics from ALS, PSP, FTD, PD, MSA, AD, and CJD were analysed to assess shared genetic architecture at *MOBP*, including pairwise SNP-level correlations and colocalisation using COLOC. Pairwise correlations between SNP-level GWAS *p*-values across diseases within the *MOBP* region (±1 Mb around the gene) were calculated to evaluate the extent of shared genetic signal. Colocalisation analysis using COLOC was performed to evaluate whether two association signals are consistent with a shared causal variant within a defined genomic region. 2) Fine mapping using the SuSiE model, which identifies credible sets of putative causal SNPs by modelling associations as the sum of multiple independent effects, was also performed. 3) Follow-up analyses integrating DNA methylation QTLs (bulk brain tissue) and gene expression QTLs (oligodendrocyte-specific) with disease-associated variants at *MOBP*. Follow up of mQTL signal in a frontal cortex white matter DNA methylation cohort rs1768208, previously linked to PSP risk, was genotyped in additional samples overlapping with frontal lobe methylation data. *MOBP*, myelin-associated oligodendrocyte protein; ALS, amyotrophic lateral sclerosis; PSP, progressive supranuclear palsy; FTD, frontotemporal dementia; AD, Alzheimer’s disease; MSA, multiple system atrophy; PD, Parkinson’s disease; CJD, Creutzfeldt-Jakob disease; GWAS, genome-wide association study; SNP, single-nucleotide polymorphism; mQTL, methylation quantitative trait loci; eQTL, expression quantitative trait loci.

To assess the effect of rs1768208 on DNA methylation, we analysed frontal lobe white-matter methylation data generated previously from control, MSA, PD, and PSP brain tissue (31) (Supplementary Table S1). Following standard pre-processing, quality control, and normalisation carried out by Murthy et al.(31), adjusted M-values, i.e. logit transformation of beta-values, were obtained for samples overlapping those successfully genotyped at rs1768208. These values were used to test for methylation differences in relation to rs1768208 genotype (presence/absence of the risk allele (T)). A schematic overview of the methodological framework if shown in Figure 1.

## Results

### Colocalisation analysis reveals shared *MOBP* genetic risk variants between ALS and PSP

**Fig. 2 |:**
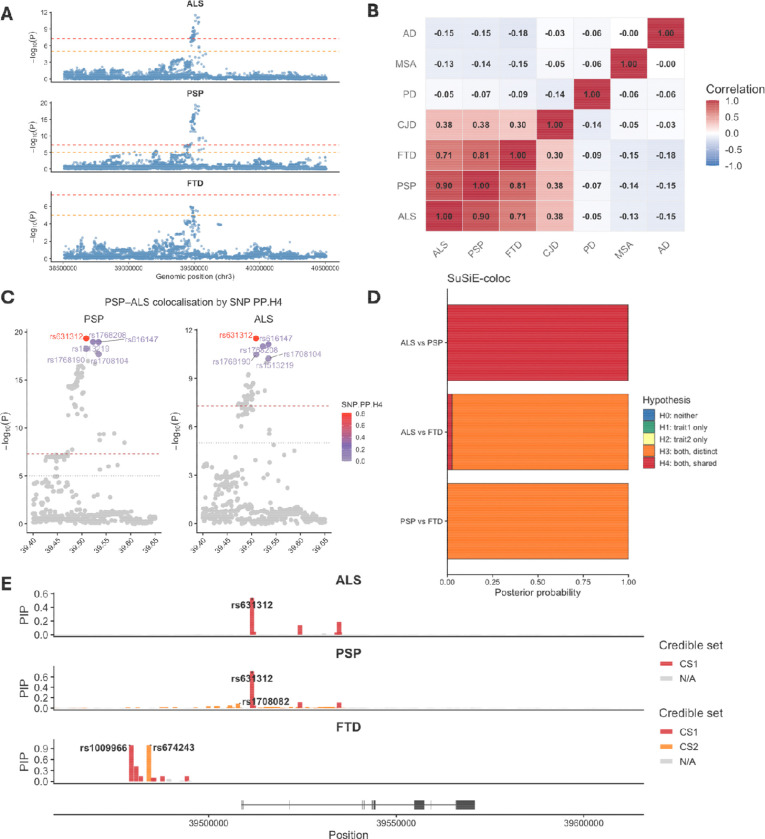
Genetic architecture and cross-disease colocalisation of the *MOBP* locus across neurodegenerative diseases. A) Regional Manhattan plots for PSP, ALS and FTD across a ±1 Mb window around the *MOBP* locus. Points represent single SNP associations, plotted as −log10(P). Red and orange dashed lines indicate genome-wide significance (P = 5×10 ) and suggestive significance (P = 1×10 ), respectively. B) Pairwise correlation heatmap of GWAS −log10(P) values across the *MOBP* region. C) Zoomed association plots for PSP and ALS around the *MOBP* peak, with SNPs coloured by their SNP-level posterior probability of belonging to the shared H4 component from COLOC.ABF (SNP.PP.H4). D) SuSiE-coloc posterior probabilities for disease-pair comparisons at *MOBP.* E) *Regional fine-mapping plots* showing SuSiE-derived credible sets for PSP, ALS, and FTD across the *MOBP* locus. SNPs are coloured by credible set membership: CS1 (primary credible set), CS2 (secondary credible set), or non-credible set (N/A). ALS, amyotrophic lateral sclerosis; PSP, progressive supranuclear palsy; FTD, frontotemporal dementia; AD, Alzheimer’s disease; MSA, multiple system atrophy; PD, Parkinson’s disease; CJD, Creutzfeldt-Jakob disease; SNP, single-nucleotide polymorphism; PP, posterior probability.

To explore the genetic landscape surrounding *MOBP* across multiple neurodegenerative disorders, we examined a ±1 Mb window around the locus using summary statistics from seven GWAS datasets: AD(24), PSP (9), FTD(21), ALS(22), PD (26), MSA (23), and CJD (25). Only ALS and PSP exhibited a clear, highly significant association peak at *MOBP* (Figure 2A, Supplementary Figure 1A), with several SNPs surpassing both genome-wide significance (P < 5 × 10 ) and the suggestive threshold (P < 1 × 10 ). FTD showed several variants reaching suggestive significance but none passing the genome-wide threshold, possibly reflecting smaller sample sizes and FTD clinicopathological heterogeneity. None of the other disorders displayed evidence of genetic association at this locus.

To determine whether different diseases share the same underlying *MOBP* risk-associated variant(s), we next quantified pairwise similarity across all SNPs in the region. ALS, PSP, and FTD showed strong positive pairwise correlations (r = 0.70–0.90, p < 1 ×10^−200^), suggesting substantial overlap in disease-associated genetic architecture at the *MOBP* locus (Figure 2B). CJD displayed moderate correlation with these three disorders (r = 0.30–0.38, p < 0.001), indicating a weaker but detectable shared component. All other disease pairs showed weak or no correlation, consistent with the absence of significant association. Together, these results demonstrate that ALS and PSP share the most pronounced and consistent genetic signal at *MOBP*, with FTD potentially showing a weaker overlap.

Given that ALS, PSP and FTD were the only diseases showing notable genetic associations at the *MOBP* locus, we prioritised detailed investigation of their relationships. We used the COLOC framework to quantify whether pairs of diseases shared the same causal genetic variant at *MOBP*. We observed very strong evidence of genetic colocalisation between ALS and PSP, with PP.H4 = 0.997 (PP.H4: both diseases are associated and share a single causal variant) (Supplementary Figure 1B), indicating that these diseases almost certainly share the same causal variant at *MOBP*. In contrast, ALS vs FTD and PSP vs FTD showed minimal support for colocalisation (PP.H4 = 0. 28 and 0.31, respectively) (Supplementary Figure 1B). For both comparisons, most of the posterior probability was assigned to PP.H3, suggesting that although FTD exhibits some signal in this region, its causal variant(s) are distinct from those driving ALS and PSP (PP.H3 = 0.69 and 0.68 for FTD vs ALS and PSP, respectively). SNP-level posterior probabilities identified a small number of variants with high PP.H4 that coincided with the association peak in both ALS and PSP, consistent with a shared signal at the *MOBP* locus (Figure 2C). In contrast, for FTD, SNPs with higher PP.H4 did not correspond to the top FTD-associated variants (Supplementary Figure 1C), supporting a distinct underlying signal. These results indicate that *MOBP* harbours a shared ALS–PSP genetic risk mechanism, whereas FTD shows a genetically separate architecture within the same locus.

### Fine mapping of *MOBP* locus confirms overlap between PSP and ALS but not FTD

As GWASs often identify broad association peaks containing many SNPs in linkage disequilibrium (LD), fine-mapping methods are required to resolve these peaks and identify which specific variants are most likely to be causal. By applying SuSiE to ALS, PSP, and FTD summary statistics, we aimed to identify the likely causal SNP(s) driving association in each disease and determine whether ALS and PSP share the same fine-mapped causal SNP(s), as well as establishing whether the FTD signal is indeed independent. We applied COLOC.SuSiE to assess colocalisation using fine-mapped posterior probabilities rather than single-SNP signals (Figure 2D). The results mirrored and strengthened the COLOC.ABF findings, with high PP.H4 for ALS-PSP (0.99) and low PP.H4 for ALS-FTD and PSP-FTD (PP.H4 = 5.3 × 10^−^ and 1.7 × 10^−1^, respectively). PSP resolved into two credible sets, with a primary CS1 centred on rs631312 and a smaller secondary CS2. ALS showed a single credible set (CS1) comprising six SNPs, four of which overlapped with PSP CS1 (rs631312, rs1768208, rs616147, rs1768190), supporting a shared fine-mapped signal. The lead SNP in both diseases was rs631312 (chr3:39,508,968 bp (GRCh37)), which had the highest posterior inclusion probability (PSP PIP = 0.70; ALS PIP = 0.54). In contrast, FTD produced a single, non-overlapping credible set located upstream of the ALS/PSP signal (top SNP chr3:39,473,591), supporting an independent genetic association at the *MOBP* locus (Figure 2E).

### Genetic associations with *MOBP* methylation links ALS and PSP

**Fig. 3 |:**
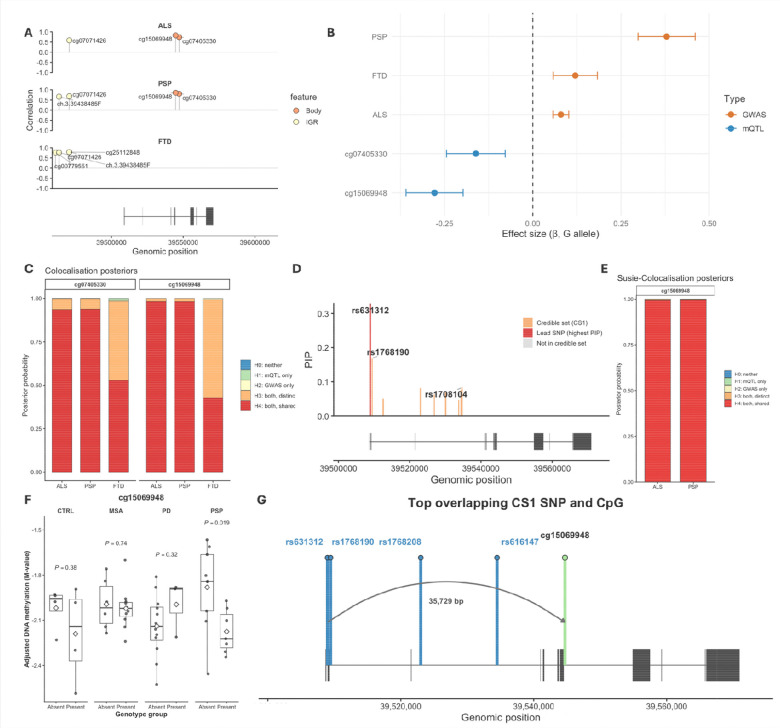
Genetic-epigenetic colocalisation and fine-mapping of mQTLs at the *MOBP* locus. A) Correlation between mQTL and GWAS signals across top CpG sites within the *MOBP* locus for ALS, PSP, and FTD. Each point represents a CpG site, plotted by genomic position. Height of lollipop point represents Pairwise correlation of GWAS −log10(P) against mQTL -log10(P) values across the *MOBP* region for ALS, PSP and FTD. CpGs are annotated and coloured by genomic feature. The gene structure for *MOBP* is shown below. B) Effect size estimates (β ± 95% CI) for rs631312 across disease GWAS and *MOBP* mQTL datasets, harmonised to the risk G allele. C) COLOC posterior probabilities for the top disease–CpG pairs ranked by H4 (shared causal variant). Bars show posterior support for each hypothesis: H0 (no association), H1 (disease only), H2 (CpG only), H3 (distinct causal variants), and H4 (shared causal variant). (D) SuSiE fine-mapping of the cg15069948 mQTL locus. Posterior inclusion probabilities (PIP) for SNPs across the *MOBP* region are shown, with CS1 SNPs highlighted in orange, and the lead SNP highlighted in red and labelled. The *MOBP* gene structure is shown below, with exons indicated as black boxes. E) SuSiE-coloc posterior probabilities between cg15069948 and disease GWAS datasets. Bars show posterior support for each hypothesis as in A. F) Frontal lobe white matter DNA methylation levels at cg15069948 across neurodegenerative disease groups, stratified by SNP risk allele presence. Differences in methylation between genotype groups were assessed using two-sample t-tests within each disease group. Boxplots show delta M-values for control, MSA, PD and PSP samples, with statistical comparisons (p-values) indicated (G) Genomic schematic of overlapping CS1 SNPs between ALS and PSP at the *MOBP* locus (blue lollipops) and the cg15069948 CpG site (green lollipop). *MOBP* exons are shown in black, and distance between the lead SNP (rs631312) and the CpG is indicated. ALS, amyotrophic lateral sclerosis; PSP, progressive supranuclear palsy; FTD, frontotemporal dementia; MSA, multiple system atrophy; PD, Parkinson’s disease; SNP, single-nucleotide polymorphism; PP, posterior probability; mQTL, methylation quantitative trait loci; CS, credible set.

Given our previous finding of aberrant DNA methylation at the *MOBP* locus in neurodegeneration (2,17), we next investigated whether genetic variants associated with ALS, PSP, or FTD colocalise with methylation quantitative trait loci (mQTLs) in human brain tissue. This allowed us to identify CpGs whose methylation levels are most strongly influenced by the disease genetic architecture. As with the GWAS–GWAS comparisons, we quantified the similarity of association signals across all SNPs in the region between GWAS and mQTL datasets. Several CpGs showed strong positive correlations with disease GWAS signals. The strongest correlations between ALS and PSP GWAS signals and mQTLs were observed at cg15069948 and cg07405330, both located within the *MOBP* gene body (cg15069948: r = 0.83 and 0.86, p < 1×10^−200^; cg07405330: r = 0.74 and 0.80, p < 1×10^−200^) for ALS and PSP, respectively) (Figure 3A, Supplementary Table S2). The risk allele (G) of the lead disease SNP for ALS and PSP identified through fine-mapping (rs631312), was associated with decreased methylation at both top correlated CpG sites (cg15069948 and cg07405330) (Figure 3B). At FTD, distinct CpGs showed the highest correlation with disease, with the top signal at cg06598794 (r = 0.82, p < 1×10^−200^), and highly correlated CpGs were predominantly located in the promoter region (Figure 3A).

We then applied COLOC.ABF to test for colocalisation between mQTLs at all CpG sites and GWAS signals for each of the three diseases. This revealed a small number of CpGs showing high PP.H4 (shared causal variant between methylation changes and disease risk) (Supplementary Table S3), with cg15069948 emerging as the strongest candidate across ALS and PSP (PP.H4 = 0.982 for both comparisons, Figure 3B). This indicates a 98% probability that ALS and PSP share a single causal variant with the mQTL controlling methylation at cg15069948. We did not see such high colocalisation with this CpG with the FTD GWAS (PP.H4 = 0.43, Figure 3C). Although several CpGs showed strong pairwise correlation between FTD GWAS and mQTL signals, none demonstrated strong evidence of colocalisation (Supplementary Table S3).

**Fig. 4 |:**
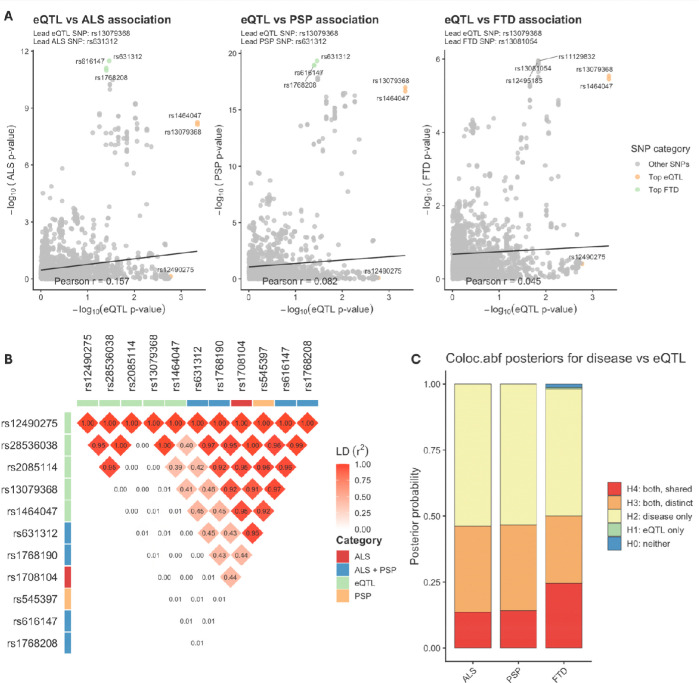
Oligodendrocyte eQTLs at the *MOBP* locus show weak correspondence with ALS, FTD, and PSP genetic risk **A)** Scatterplots comparing SNP-level eQTL association strength (−log *p*) with GWAS association strength for ALS, PSP and FTD across the *MOBP* locus (±1 Mb). Each point represents a shared SNP between datasets. Linear regression lines and Pearson correlation coefficients are shown. Lead SNPs (highest (−log *p*)) for each dataset are indicated. Top 3 SNPs for each dataset are coloured orange (eQTLs) and green (disease), and don’t seem to overlap. B) Pairwise linkage disequilibrium (LD; *r*^2^) between SNPs within the *MOBP* locus is shown as a triangular heatmap. Each diamond represents the LD between a pair of variants, with colour intensity indicating the strength of correlation (white to red corresponding to *r*^2^ = 0 to 1). Numerical values within each cell denote the corresponding *r*^2^. Variants are annotated by association category, indicated by coloured bars along the axes: SNPs associated with ALS (red), PSP (orange), shared ALS+PSP signal (blue), and eQTLs (green). **C)** COLOC.ABF posterior probabilities for eQTL–GWAS colocalization reveal no evidence of colocalisation. ALS, amyotrophic lateral sclerosis; PSP, progressive supranuclear palsy; FTD, frontotemporal dementia; SNP, single-nucleotide polymorphism; PP, posterior probability; eQTL, expression quantitative trait loci; ABF, approximate Bayesian factor; LD, linkage disequilibrium.

Fine-mapping of the cg15069948 mQTL revealed a single credible set (CS1) centred on the *MOBP* locus (Figure 4D). CS1 contained nine SNPs, with rs631312 showing the highest inclusion probability (PIP = 0.33) (Figure 4B). To assess whether the mQTL credible set overlaps biologically with disease-associated SNPs, we compared the SuSiE-derived mQTL CS1 with the fine-mapped credible sets from ALS, PSP, and FTD. There was substantial overlap between the mQTL CS1 and the ALS and PSP CS1 sets, with 6/6 shared SNPs for ALS and 4/4 for PSP. These shared variants include rs631312, rs1768208, rs1768190 and rs616147, the SNPs that also drive the strongest GWAS associations in both diseases.

Whereas COLOC.ABF already indicated strong support for a shared causal variant (PP.H4 = 0.99 for both ALS–mQTL and PSP–mQTL comparisons), COLOC.SuSiE provided even stronger mechanistic evidence by demonstrating that the same specific SuSiE credible set accounts for both the mQTL signal and the ALS/PSP GWAS signals (Figure 4E). In both ALS and PSP, the majority of posterior mass-mapped onto the same mQTL CS1 SNPs, confirming that these variants jointly explain the methylation and disease associations.

In contrast, FTD showed no credible-set overlap with the mQTL (PP.H4 = 0.16; zero shared SNPs), with posterior support instead supporting evidence for both traits being associated, but with different causal variants.

To complement the analysis of genetic variation at *MOBP* and any associated DNA methylation variation across neurodegenerative diseases, we undertook genotyping of the SNP rs1768208 (which is high LD with the lead SNP rs631312) to investigate any effects of genotype on DNA methylation in samples we had previously generated DNA methylation profiles of frontal lobe white matter across PSP, MSA and PD (31). The same CpG identified above as being an mQTL with strong association with PSP and ALS (cg15069948) showed nominally significant differences in methylation within PSP samples between presence and absence of the risk allele (P = 0.019) (Figure 4F), but not in the other neurodegenerative diseases. The direction of effect, a decrease in DNA methylation being associated with the presence of the risk allele (rs1768208-T), is in accordance with the direction of effect from the mQTL summary statistics. The finding of cg15069948 as linked to PSP through two distinct analyses highlights its biological relevance to linked genetic and epigenetic modulation of *MOBP.*

### eQTLs Show Limited Evidence of Shared Causal Variants at MOBP

We next assessed whether oligodendrocyte-specific eQTLs derived from single-nucleus RNA-seq of the frontal cortex (28) at the *MOBP* locus colocalise with ALS, FTD, or PSP risk SNPs. As an initial exploration, we compared eQTL and GWAS p-values across SNPs within ±1 Mb of *MOBP*. In contrast to the strong SNP-wise correlations observed for mQTLs, eQTL–GWAS correlations were uniformly weak (Figure 5A).

To further investigate this, we examined local linkage disequilibrium (LD) structure across all variants (Figure 5B). This revealed distinct eQTL clusters largely independent of disease-associated variants: one showing moderate LD (r^2^ ≈ 0.4–0.5) and another with minimal LD (r^2^ ≈ 0–0.1). Even the moderately correlated cluster did not strongly overlap with lead disease SNPs, and most eQTLs were effectively independent of ALS-, FTD-, and PSP-associated variants.

Consistent with this, COLOC.ABF analysis using eQTL summary statistics (N = 192) showed little evidence of a shared causal variant (PP.H4 = 0.14 for ALS/PSP; 0.25 for FTD; Figure 5C), with H2/H3 models strongly favoured. SuSiE likewise identified no credible sets, and posterior inclusion probabilities were uniformly low.

## Discussion

Here, we investigated genetic and epigenetic variation at the *MOBP* locus across multiple neurodegenerative diseases, revealing both shared and disease-specific mechanisms. Analysis of GWAS summary statistics identified significant associations in ALS, PSP, and FTD. Notably, ALS and PSP showed strong overlap in lead risk SNPs and high posterior probabilities of colocalisation, indicating a shared underlying causal variant. Bayesian fine-mapping further supported this convergence, with credible sets in ALS and PSP highlighting a largely overlapping set of variants within the *MOBP* locus. In contrast, FTD demonstrated weak evidence of colocalisation with ALS and PSP, and fine-mapping localised FTD-associated variants to a region upstream of the *MOBP* transcription start site, suggesting a distinct regulatory mechanism. mQTL analyses further highlighted cg15069948 as a key CpG site linking genetic risk to DNA methylation, acting as a significant mQTL for the shared ALS and PSP top SNPs. However, we did not observe robust eQTLs linking disease-associated variants to *MOBP* gene expression in available brain datasets. Overall, these findings suggest that *MOBP* eQTL variation in oligodendrocytes is unlikely to explain disease risk, and that the underlying genetic mechanisms at this locus are more consistent with mQTL regulation.

There is increasing recognition of the importance of oligodendrocytes and myelin involvement in neurodegeneration(32–37). Many neurodegenerative diseases exhibit well described oligodendrocyte pathology, for example in MSA (38), where they contain characteristic glial cytoplasmic inclusions composed of aggregated α-synuclein. Primary tauopathies such as PSP and corticobasal degeneration (CBD) also exhibit prominent OLG pathology, with tau accumulation forming coiled bodies within these cells (39,40). In ALS, where loss of motor neurons is the major pathological hallmark of disease, there is also growing evidence for oligodendrocyte involvement(41,42). The recurrent identification of myelin-associated genes such as *MOBP* across multiple neurodegenerative diseases further underscores the importance of oligodendrocyte and myelin biology, highlighting the need to better understand their contribution to disease mechanisms.

Prior epigenetic studies have reported promoter hypermethylation of *MOBP* in MSA, accompanied by reduced gene expression, implicating DNA methylation as a potential regulatory mechanism at this locus (2,17,18). However, we did not observe evidence of genetic association linking *MOBP* to MSA, suggesting that epigenetic dysregulation at this locus in MSA may occur independently of inherited genetic variation. Altered DNA methylation at *MOBP* in MSA provided a strong rationale to investigate whether disease-associated genetic variation at *MOBP* converges on altered DNA methylation across neurodegenerative diseases, and whether such epigenetic changes may reflect shared or disease-specific regulatory pathways. Our findings indicate that while ALS and PSP share a common genetic risk signal at *MOBP* that colocalises with DNA methylation, FTD likely involves a distinct genetic and regulatory contribution within the same locus. Together, these results suggest that *MOBP* represents a convergent gene implicated across neurodegenerative diseases, but through different mechanisms, highlighting a nuanced interplay between genetics and epigenetics in disease risk.

The absence of a detectable eQTL at the *MOBP* locus raises the possibility that DNA methylation may influence gene regulation through mechanisms other than steady-state gene expression. DNA methylation within gene bodies and regulatory regions has been implicated in the modulation of transcriptional dynamics, including alternative splicing and exon inclusion or exclusion, rather than overall transcript abundance(43,44). In this context, disease-associated methylation changes at *MOBP* may affect isoform usage, transcript stability, or transcriptional elongation in a cell-type-specific manner. Such mechanisms would not be captured by standard eQTL analyses but could nonetheless have important functional consequences for oligodendrocyte biology and myelin integrity. Our previous work shows that in PSP white matter tissue (highly enriched for oligodendrocytes) there is an increase in MOBP protein expression and a shift toward larger isoforms(2), supporting a role for altered transcript usage in disease, resulting in altered ratios of protein isoforms.

An important question arising from our analyses is why genetic association at the *MOBP* locus appears to cluster across specific neurodegenerative diseases, while being absent or weaker in others. One potential explanation relates to shared underlying pathological processes, particularly the nature of proteinaceous inclusions that define each disease. ALS and PSP show overlapping genetic signals at *MOBP*, and while PSP is classed as a primary tauopathy, ALS, although classically characterised by TDP-43 pathology, has also been associated with altered tau metabolism(45). We also saw a suggestively significant signal at the *MOBP* locus in FTD in a mixed FTD-TDP and FTD-tau cohort(21). Supporting a potential link to tau pathology, the PSP risk allele (T) at rs1768208 has been associated with increased tau thread pathology and phosphorylated tau-positive coiled bodies in oligodendrocytes (15). However, a purely tau associated explanation is unlikely, as we did not see any colocalization at *MOBP* in AD, despite its classification as a tauopathy. Although *MOBP* has previously been linked to AD, rs1768208 (in high LD with our identified lead SNP at ALS/PSP) was only associated in APOE ε4 positive subjects(13). *APOE*, the strongest genetic risk factor for AD, has recently been implicated in oligodendrocyte dysfunction, with APOE ε4 associated with reduced expression of myelin-related genes and impaired cholesterol handling in oligodendrocytes (46). Together, these observations raise the possibility that genetic variation at *MOBP* may exacerbate oligodendrocyte vulnerability in specific pathological contexts, and that additional factors such as *APOE* genotype may be required for genetic risk at this locus to manifest in AD.

We also considered whether the association was linked to the amount of oligodendrocyte pathology reported in these diseases. As described above, a pathological hallmark of PSP is cytoplasmic inclusions in oligodendrocytes presenting as coiled bodies (13,47) and there is growing evidence as to the involvement of oligodendrocytes in ALS (41,42). In MSA, a disease in which the primary cell type affected by pathology is oligodendrocytes, one would expect that if it was the case that the genetic association was linked to degree of oligodendrocyte pathology, we would see a stronger genetic signal at *MOBP.* Nevertheless, in MSA *MOBP* expression seems to be dysregulated via hypermethylation at the promoter region. Altogether, this suggests that different mechanisms might be affecting the dysregulation of this locus and disease risk differently across diseases, implicating DNA methylation only in MSA and genetic variation influencing DNA methylation in PSP and ALS.

Whilst our analysis into *MOBP* across neurodegenerative diseases has provided important insights, there are some limitations to discuss. As with any analysis utilising publicly available data, we rely upon quality and completeness of datasets. Heterogeneity, co-pathologies and misdiagnosis can confound GWAS analyses, leading to apparent associations that may not be disease-specific while reducing power to detect true genetic effects in specific diseases. Additionally, we cannot exclude the possibility of the smaller GWAS being underpowered or brain region specific effects on QTLs.

In this work we have carried out analyses to investigate genetics, mQTLs and eQTLs at *MOBP* across neurodegenerative diseases, to further understanding of the involvement of this gene in pathology. We have found for the first time a shared locus of genetic association within *MOBP* between ALS and PSP, which is also associated with DNA methylation. This seems to be distinct from the FTD genetic signal and the promoter region hypermethylation in MSA. Elucidating how genetic variation at *MOBP* converges between diseases, and on DNA methylation, provides a mechanistic bridge between inherited risk and downstream gene dysregulation, and identifies epigenetic processes as potential therapeutic targets given their dynamic and potentially reversible nature(48). This work has provided key insights into the importance of *MOBP* in neurodegeneration and established a foundation for future research into this gene.

## Supplementary Material

Supplement 1

Supplement 2

## Figures and Tables

**Table 1.** Description of the disease GWAS used to investigate genetic associations at *MOBP* across neurodegenerative diseases

Disease	Sample Size	Reference
PSP	2,779 cases (2,595 neuropathologically-confirmed), 5,584 controls	(9)
FTD	4,685 cases, 15,308 controls	(21)
ALS	29,612 cases, 122,656 controls	(22)
MSA	888 cases, 7,128 controls	(23)
AD	111,326 clinically diagnosed/’proxy’ cases, 677,663 controls	(24)
CJD	4,110 cases, 13,569 controls	(25)
PD	37,700 cases, 18,600 proxy cases, 1,400,000 controls	(26)

PSP: progressive supranuclear palsy, FTD: frontotemporal dementia, ALS: amyotrophic lateral sclerosis, LBD: Lewy body dementia, MSA: multiple system atrophy, AD: Alzheimer’s disease, CJD: Creutzfeldt–Jakob disease, PD: Parkinson’s disease.

## Data Availability

ALS, MSA, AD, CJD and PD GWAS summary statistics were accessed through the GWAS Catalog (accession numbers GCST90027164, GCST90406924, GCST90027158, GCST90001389 and GCST009325 respectively). FTD GWAS summary statistics were accessed from the UCL Research Data Depository (https://doi.org/10.5522/04/25600692.v1.). eQTL summary statistics were accessed through https://zenodo.org/records/7276971. mQTL summary statistics were provided by the study authors (27).

## Code Availability

All code used for analysis will be made available upon publication in a publicly accessible GitHub repository: https://github.com/CBettencourtLab/MOBPAnalysis.
