# Supplementary material for: Cross-disease genetic and epigenetic architecture of the MOBP locus shows convergence in ALS-PSP
Source: bioRxiv. 2026 Mar 27:2026.03.25.714147. Preprint. [Version 1] doi: 10.64898/2026.03.25.714147 (PMC13041890; doi:10.64898/2026.03.25.714147)
Supplement: Supplement 2 [file media-2.docx]

# **Supplementary Figures:** Cross-disease genetic and epigenetic architecture of the *MOBP* locus shows convergence in ALS-PSP

Katherine Fodder^1^, Megha Murthy^2^, Rohan de Silva^2,3^, Towfique Raj^4,5,6,7^, Kurt Farrell^5,6,7,8,9,10^, Jack Humphrey^4,5,6,7^**^*^** Conceição Bettencourt^1^**^*^**

1. Department of Neurodegenerative Disease, UCL Queen Square Institute of Neurology, London, UK

2. Department of Clinical and Movement Neurosciences, UCL Queen Square Institute of Neurology, London, UK

3. Reta Lila Weston Institute, UCL Queen Square Institute of Neurology, London, UK

4. Department of Genetics and Genomic Sciences, Icahn Genomics Institute, Estelle and Daniel Maggin Department of Neurology, Icahn School of Medicine at Mount Sinai, New York, NY, USA.

5. Nash Family Department of Neuroscience, Icahn School of Medicine at Mount Sinai, New York, NY, USA

6. Ronald M. Loeb Center for Alzheimer’s Disease, Icahn School of Medicine at Mount Sinai, New York, NY, USA

7. Friedman Brain Institute, Icahn School of Medicine at Mount Sinai, New York, NY, USA

8. Department of Pathology, Icahn School of Medicine at Mount Sinai, New York, NY, USA

9. Department of Artificial Intelligence & Human Health, Icahn School of Medicine at Mount Sinai, New York, NY, USA

10. Neuropathology Brain Bank & Research CoRE, Icahn School of Medicine at Mount Sinai, New York, NY, USA

*Corresponding Authors:

Jack Humphrey, PhD
Icahn School of Medicine at Mount Sinai
1 Gustave Levy Plaza
New York 10029
[jack.humphrey@mssm.edu](mailto:jack.humphrey@mssm.edu)

Conceição Bettencourt, PhD
Department of Neurodegenerative Disease
UCL Queen Square Institute of Neurology
1 Wakefield Street
London WC1N 1PJ
United Kingdom
[c.bettencourt@ucl.ac.uk](mailto:c.bettencourt@ucl.ac.uk)


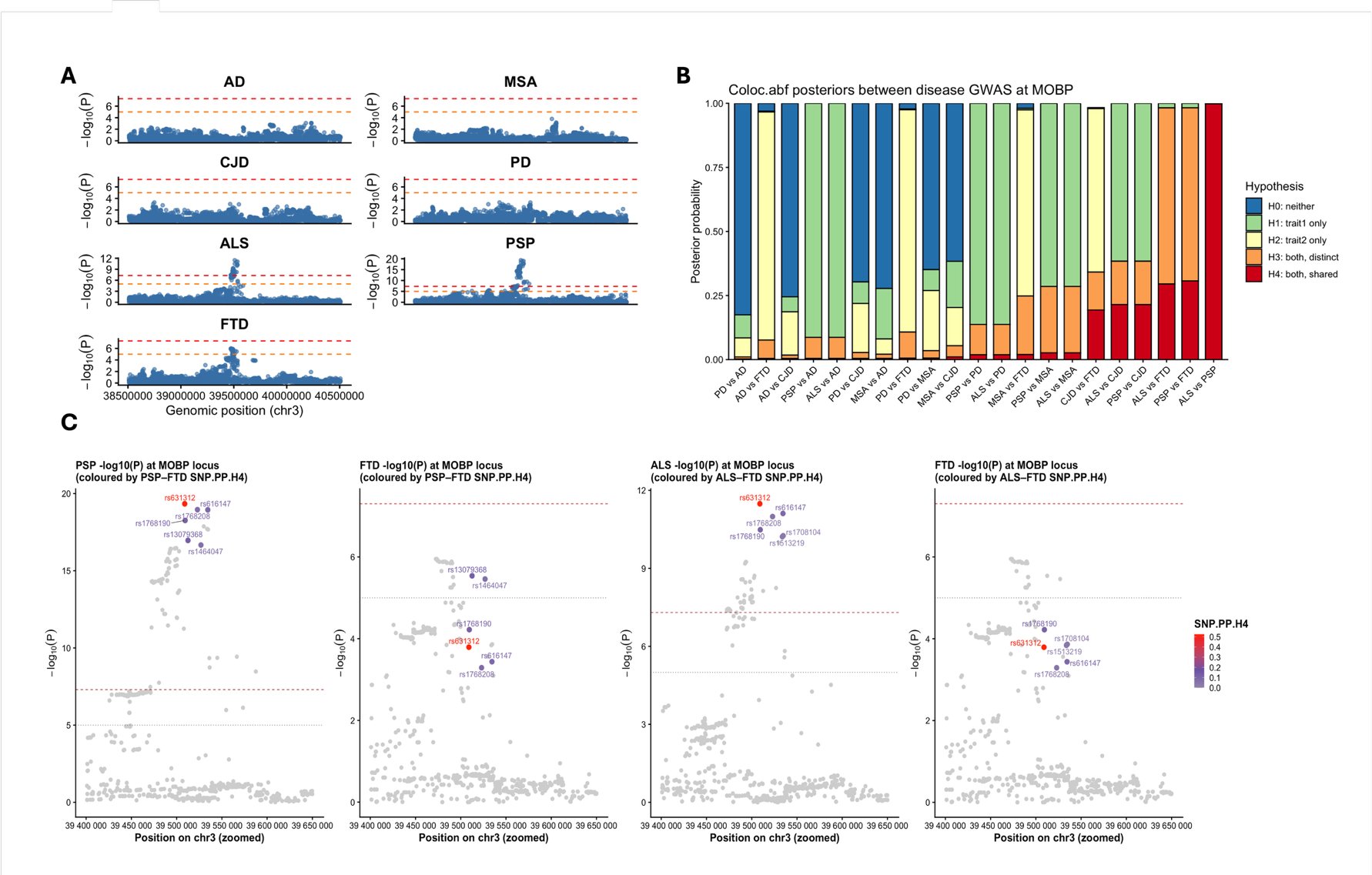


**Supplementary Fig. 1 | Genetic architecture and cross-disease colocalisation of the *MOBP* locus across neurodegenerative diseases*.***

A) Regional Manhattan plots for AD, MSA, CJD, PD, ALS, PSP and FTD across a ±1 Mb window around the *MOBP* locus (chr3). Points represent single SNP associations, plotted as –log10(P). Red and orange dashed lines indicate genome-wide significance (P = 5×10⁻⁸) and suggestive significance (P = 1×10⁻⁵), respectively. B) Pairwise correlation heatmap of GWAS –log10(P) values across the *MOBP* region. C) COLOC.ABF posterior probability distribution for all pairwise GWAS comparisons at the MOBP locus. Bars show posterior probabilities for hypotheses H0–H4: H0 = neither trait associated; H1 = trait 1 only; H2 = trait 2 only; H3 = both traits associated but with distinct causal variants; H4 = both traits share a causal variant. C) Zoomed association plots for PSP, ALS and FTD around the *MOBP* peak, with SNPs coloured by their SNP-level posterior probability of belonging to the shared H4 component from COLOC.ABF (SNP.PP.H4). ALS, amyotrophic lateral sclerosis; PSP, progressive supranuclear palsy; FTD, frontotemporal dementia; AD, Alzheimer’s disease; MSA, multiple system atrophy; PD, Parkinson’s disease; CJD, Creutzfeldt-Jakob disease; SNP, single-nucleotide polymorphism; PP, posterior probability.
